# Feasibility of using acceleration-derived jerk to quantify bimanual arm use

**DOI:** 10.1186/s12984-020-0653-2

**Published:** 2020-03-16

**Authors:** Ying-Chun (Preston) Pan, Brianna Goodwin, Emily Sabelhaus, Keshia M. Peters, Kristie F. Bjornson, Kelly L. D. Pham, William Walker, Katherine M. Steele

**Affiliations:** 1 Department of Bioengineering, University of Washington, Seattle, WA USA; 2 Seattle Children’s Hospital, Seattle, WA USA; 3 Department of Mechanical Engineering, University of Washington, Seattle, WA USA; 4 Department of Pediatrics, University of Washington, Seattle Children’s Research Institute, Seattle, WA USA; 5 Department of Rehabilitation Medicine, University of Washington, Seattle, WA USA

**Keywords:** Rehabilitation, Cerebral palsy, Jerk, Activity count

## Abstract

**Background:**

Accelerometers have become common for evaluating the efficacy of rehabilitation for patients with neurologic disorders. For example, metrics like use ratio (UR) and magnitude ratio (MR) have been shown to differentiate movement patterns of children with cerebral palsy (CP) compared to typically-developing (TD) peers. However, these metrics are calculated from “activity counts” – a measure based on proprietary algorithms that approximate movement duration and intensity from raw accelerometer data. Algorithms used to calculate activity counts vary between devices, limiting comparisons of clinical and research results. The goal of this research was to develop complementary metrics based on raw accelerometer data to analyze arm movement after neurologic injury.

**Method:**

We calculated jerk, the derivative of acceleration, to evaluate arm movement from accelerometer data. To complement current measures, we calculated jerk ratio (JR) as the relative jerk magnitude of the dominant (non-paretic) and non-dominant (paretic) arms. We evaluated the JR distribution between arms and calculated the 50th percentile of the JR distribution (JR50). To evaluate these metrics, we analyzed bimanual accelerometry data for five children with hemiplegic CP who underwent Constraint-Induced Movement Therapy (CIMT) and five typically developing (TD) children. We compared JR between the CP and TD cohorts, and to activity count metrics.

**Results:**

The JR50 differentiated between the CP and TD cohorts (CP = 0.578 ± 0.041 before CIMT, TD = 0.506 ± 0.026), demonstrating increased reliance on the dominant arm for the CP cohort. Jerk metrics also quantified changes in arm use during and after therapy (e.g., JR50 = 0.378 ± 0.125 during CIMT, 0.591 ± 0.057 after CIMT). The JR was strongly correlated with UR and MR (*r* = − 0.92, 0.89) for the CP cohort. For the TD cohort, JR50 was repeatable across three data collection periods with an average similarity of 0.945 ± 0.015.

**Conclusions:**

Acceleration-derived jerk captured differences in motion between TD and CP cohorts and correlated with activity count metrics. The code for calculating and plotting JR is open-source and available for others to use and build upon. By identifying device-independent metrics that can quantify arm movement in daily life, we hope to facilitate collaboration for rehabilitation research using wearable technologies.

## Background

Accelerometers, sensors that measure linear acceleration, have become a common tool to assess physical movement [1]. These small sensors provide objective and precise measures of motion and are ubiquitous in modern products such as smartphones. In addition to monitoring movement patterns of healthy adults [2], these sensors are also capable of quantifying motion in clinical populations [3–5]. Because of their portability, affordability, and continuous monitoring capability, accelerometers provide a quantitative adjunct to evaluating treatment efficacy and complement other in-clinic evaluations.

One clinical application of accelerometers has been to assess interventions to improve arm function after neurologic injury, such as among stroke survivors or individuals with cerebral palsy [6–12]. For these applications, accelerometer data have been used to analyze movement in terms of activity counts [13]. Activity counts have been shown to provide an accurate and repeatable measure of both the duration and intensity of activity, and have become one of the most commonly used measures for accelerometer-based rehabilitation research [14]. Accelerometers worn on each wrist have been used to calculate activity counts, which are then used to calculate common outcome measures that compare dominant and non-dominant arm use during clinical tests or daily activity. Some of the most common outcomes based on activity counts include the magnitude ratio (MR), which compares the relative intensity of movement between arms, and the use ratio (UR) which compares duration of arm use [6, 12]. These metrics have been used to evaluate function before and after interventions and can detect clinically meaningful changes in function. For example, MR and UR have been used to evaluate movement at home and in the clinic for adult stroke [15, 16] and inform the efficacy of rehabilitation [17].

While pre-processed activity counts are convenient and demonstrate less variability than raw acceleration data, they are not standardized and depend on proprietary algorithms [13]. As highlighted by Hayward and colleagues (2010), “Different brands of accelerometers have different processes for integrating the signal to produce activity counts, which are not publicly available. This inherently makes it difficult to directly compare activity counts provided by different accelerometer brands” [18]. Even for devices from the same manufacturer, significant differences in activity counts have been reported, suggesting changes in algorithms that limit longitudinal evaluations [19]. This lack of transparency not only limits the understanding of the factors that influence these outcome measures, but also hinders clinicians’ and researchers’ ability to compare measures and interpret outcomes. While commercial platforms have decreased the cost of accelerometer technology, increased access, and created user-friendly interfaces, there remains a need to develop open-source algorithms that can capture clinically relevant changes in arm function. Ideally, such algorithms could use raw accelerometer data from any device to compare across studies.

The objectives of this research were to (1) derive a metric from raw accelerometer data to quantify arm movement, (2) evaluate whether this metric can quantify bimanual arm use for a rehabilitation intervention, and (3) provide open-source code for others to use and build upon. Based upon the early research and documented limitations of evaluating movement from raw acceleration data, we focused this analysis on jerk, the time rate of change in acceleration. As early as 1985, Flash & Hogan described the coordination of arm movements with jerk, noting its advantage of capturing smoothness of movement [20], which is also commonly altered after neurologic injury [21]. Lucena and colleagues in 2017 showed the potential benefits of using jerk measured from inertial measurement units (IMUs) to evaluate bimanual arm use among stroke survivors [22]. They showed strong correlation with activity count measures; however, an IMU requires significantly more power, data storage, and cost than an accelerometer alone. In this research, we propose that jerk ratio (JR), a measure of the relative jerk between arms from wrist-worn accelerometers, is comparable to activity count measures in its ability to differentiate between children with unilateral cerebral palsy (CP) and typically-developing (TD) peers.

## Methods

To evaluate jerk and activity count measures, we analyzed previously collected and reported data from five children with unilateral CP (3 M/2F, age: 7.2 ± 0.5 years, height: 125.5 ± 9.3 cm, weight: 29.0 ± 11.7 kg, average ± one standard deviation) before and after CIMT and 5 TD peers (1 M/4F, age: 7.8 ± 1.1 years, height: 127.6 ± 8.3 cm, weight: 25.8 ± 1.8 kg) [23]. While the original study had seven children in each cohort, we excluded patients who used a walker (*n* = 1) or who were outside of the age range between seven and nine to limit age effects (*n* = 1). The function of the children with CP were classified as Gross Motor Functional Classification System (GMFCS) Levels I-III, Manual Ability Classification System (MACS) Levels II-III, and had Functional Independence Measure for children (WeeFIM) self-care scores ranging from 2 to 7 (Table 1) [24–26]. CP03 had mild dystonia.

**Table 1:** Demographics and functional scales for the cerebral palsy group

	Gender	Age	GMFCS	MACS	WeeFIM^a^
CP01	M	7	II	II	36
CP02	F	7	II	II	35
CP03	M	7	III	III	23
CP04	F	8	II	II	30
CP05	M	7	I	II	32

^a^Sum of WeeFIM self-care scores for eating, grooming, bathing, dressing (upper and lower extremity), and toileting (6 = dependence, and 42 = complete independence)

Accelerometers were placed on both wrists of the participants. Each child wore the sensors for three days during three separate time periods. The children with CP wore the sensors: 1) 1 to 2 weeks before the start of CIMT, 2) during the second week of CIMT, and 3) 6 to 8 weeks after CIMT, while the TD cohort wore the sensors during time periods temporally spaced to align with the data collections for the CP cohort. The CIMT protocol involved wearing a cast on the paretic arm for 3 weeks, with 2 h of occupational therapy focused on upper extremity function at a tertiary children’s hospital on weekdays during the treatment period. Previous research using activity count metrics from this dataset demonstrated significant differences between TD and CP cohorts, and significant changes in arm function during CIMT for the children with CP. Data were collected using the ActiGraph GT9X Link accelerometer (Actigraph, Pensacola, FL) at 100 Hz. This small and wireless tri-axial accelerometer has a dynamic range of ±8 gravitational units. We synchronized the start time of the accelerometers using the ActiLife 6 software.

Data were stored on the device and raw accelerometer data were downloaded to a local machine using ActiLife 6. We applied a fourth order Butterworth bandpass filter with cutoff frequencies at 0.25 and 2.5 Hz to our data as this filtering scheme was shown to align with human motion [27]. We also obtained activity counts calculated during one-second time epochs using ActiLife 6 as our comparison measurement. We calculated the magnitude ratio (MR) and use ratio (UR) from the activity counts. The MR is calculated by taking the natural logarithm of the ratio of the vector magnitude of activity counts from the non-dominant arm and dominant arm for each time epoch [12]. To avoid infinite values, the MR excludes time points when either arm has zero activity. The UR is calculated as the duration of activity in the non-dominant (paretic) arm over that in the dominant arm, where duration is defined as the number of time epochs with activity count magnitude greater than or equal to two [6]. For all the children with CP, the paretic arm was the child’s non-dominant arm.

To mirror these common activity count measures, we quantified upper limb movement with two outcome measures derived from jerk: jerk ratio (JR) and jerk ratio-50% (JR50). Jerk is the time rate of change of acceleration and can be discreetly estimated by taking the difference of two subsequent acceleration measurements in each direction and dividing by the change in time. JR is defined as the ratio of the magnitude of jerk of the non-dominant (ND) arm over the sum of the magnitude of ND and dominant (D) jerk:

1 $$ jerk\ ratio\ (JR)=\frac{\mid N{D}_{jerk}\mid }{\left|N{D}_{jerk}\right|+\mid {D}_{jerk}\mid } $$

Time points where |NDjerk| and |Djerk| were both zero were excluded from the analysis, although this was rare since raw acceleration measurements in g are less processed than activity counts. For a given time point, a JR close to 1 or 0 suggests mostly non-dominant or dominant arm movement, respectively. JR was calculated for the collection period and filtered using a fourth order, lowpass Butterworth filter with a cutoff frequency of 3 Hz. The rationale behind this secondary filter was to minimize noise that arises from changes in orientation. Without the magnetometer to detect device orientation, the present study assumes that jerk derived from rotational motion would both be random and high frequency.

Once JR was calculated, a probability density function (PDF) was estimated by normalizing the histogram with respect to the whole three-day collection period such that the total probability of the distribution is equal to one. In comparing PDF across collection periods, all PDFs were normalized by their maximum values. To provide a summary metric of the JR distribution, JR50 was calculated as the cumulative probability from JR = 0 to 0.5, where values over 0.5 suggest more frequent dominant arm use. Importantly, JR and JR50 are inversely related. In the case of high dominant arm use, the entire JR distribution could lie to the left of JR = 0.5 and result in a JR50 of 1.0 (100%) while the JR of individual time points might all be less than 0.5.

We quantified the consistency of JR50 by comparing JR distributions under different conditions and calculating Pearson’s correlation coefficient (*r*). Similar to JR, MR is also a distribution of time points and summarized with MR50. To compare whether there are advantages to using jerk versus acceleration, we applied the same methods (eq. 1) to the raw acceleration data to calculate an equivalent acceleration ratio (AR). We used linear regression to compare JR and AR to activity count metrics (UR and MR) for TD and CP participants, evaluating the fit with Pearson’s correlation coefficient (*r*).

## Results

**Fig. 1:**
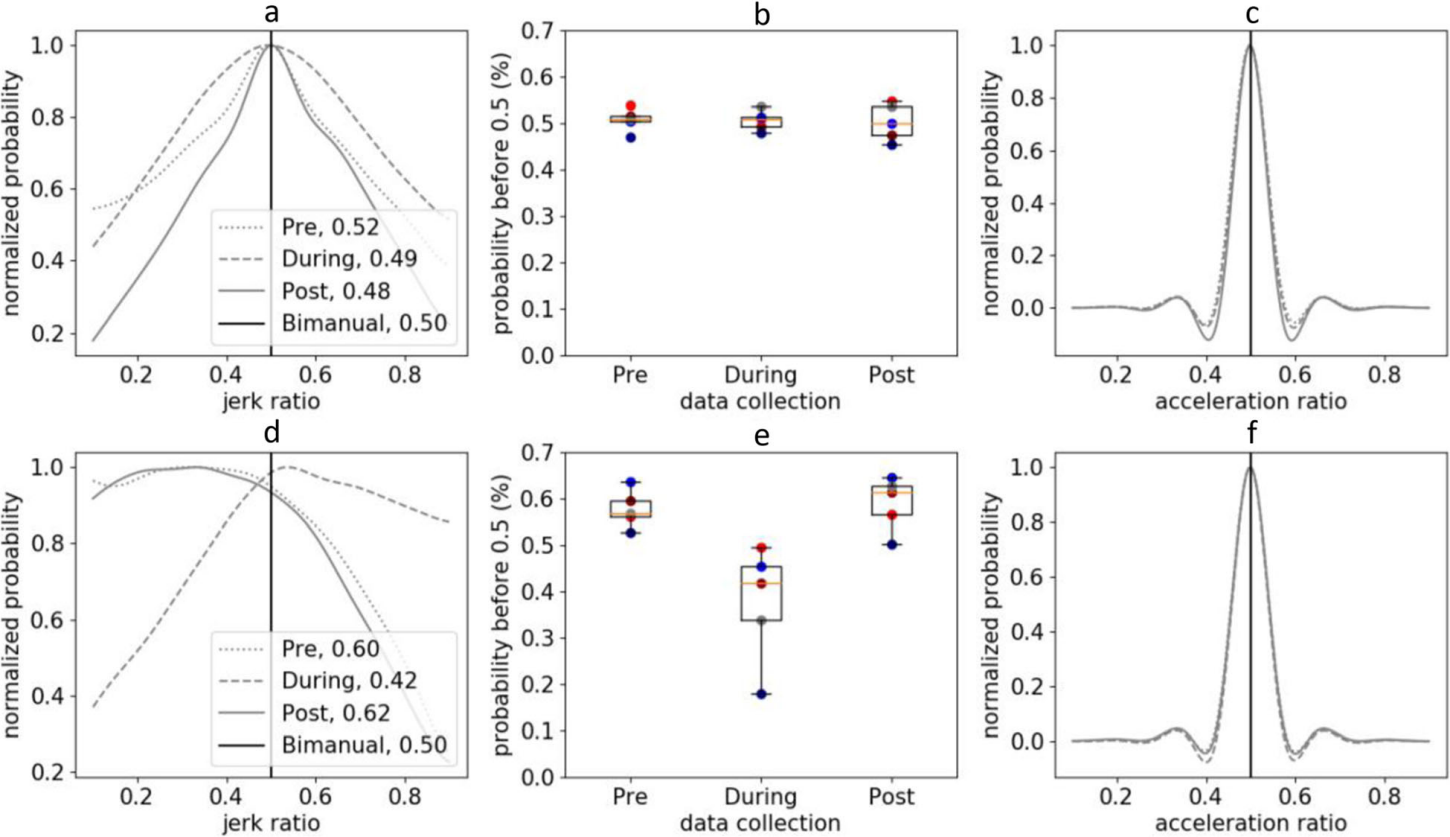
**a**: Jerk ratio histogram across all three collection periods of one TD participant. **b**: JR50 of all TD children demonstrates high similarity between participants and across collection periods. **c**: Sample distribution if acceleration was used versus jerk. **d**, **e**, **f** present the same information for the CP group. Note the asymmetric JR distribution for one child with CP (**d**), changes in JR50 with therapy, and that these differences are not detected if acceleration was used (**f**). Similar distribution plots (e.g., **a** and **d**) for all participants can be found in the Supplementary Material or generated from the open-source code. The colored circles (**b** and **e**) show JR50 for each child

For TD children, the distribution of JR during daily life was symmetric and had a median value of JR50 = 0.506 ± 0.026 (Fig. 1a). Examining the distribution of JR provides insight into the relative amount of unimanual versus bimanual activity in daily life. For TD children, this distribution indicates that there is more bimanual arm use than unimanual motion in daily life. The distribution was moderately repeatable between days and similar between TD children. The average similarity of JR distribution between collection periods was 0.945 ± 0.015. The average similarity of the JR distribution between TD children was 0.860 ± 0.137.

**Fig. 2:**
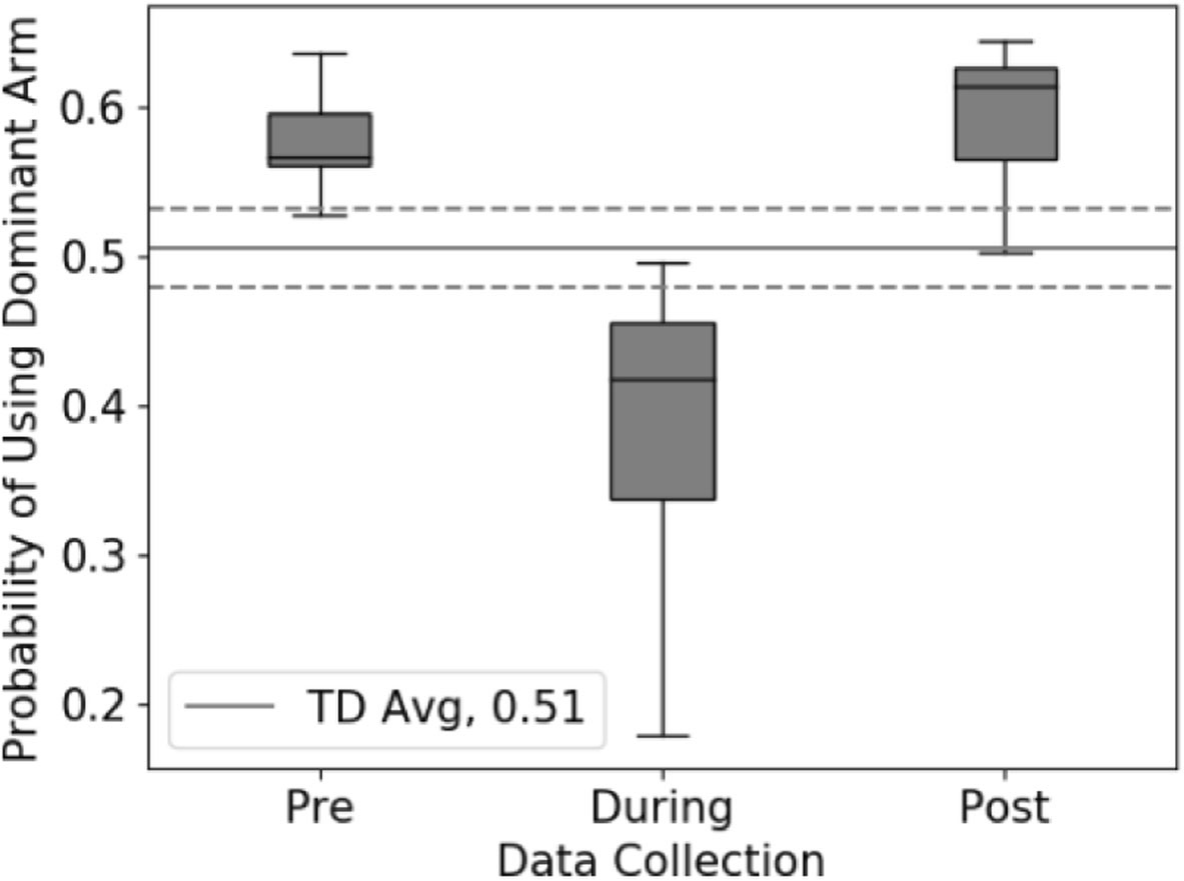
The JR50 values of all CP participants demonstrate that before and after therapy, the children with CP are more reliant on their dominant arm than TD peers. During therapy, dominant arm movement is significantly reduced due to the cast. The dotted grey lines represent the range of JR50 values across the TD cohort

As expected, the distribution of JR before therapy was asymmetric for children with CP and had a higher median value of JR50 = 0.578 ± 0.041 (Fig. 1d), indicating that they were more reliant on their dominant, non-paretic arm in daily life before CIMT. The JR distribution was similar between the children with CP, with an average similarity in JR distribution of 0.913 ± 0.069 before therapy. The JR was also sensitive to changes in arm use during therapy. The JR50 dropped during therapy to 0.378 ± 0.125, suggesting more paretic arm use during CIMT when the dominant arm was in a cast. However, the JR returned to baseline after CIMT with an average JR50 of 0.591 ± 0.057 (Fig. 2). The average similarity to the TD JR distribution was 0.726 ± 0.184 before therapy, 0.350 ± 0.643 during therapy, and 0.731 ± 0.225 after therapy. The changes in JR during treatment and compared to TD peers parallel the observations made from activity counts reported in the original analysis [23].

**Fig. 3:**
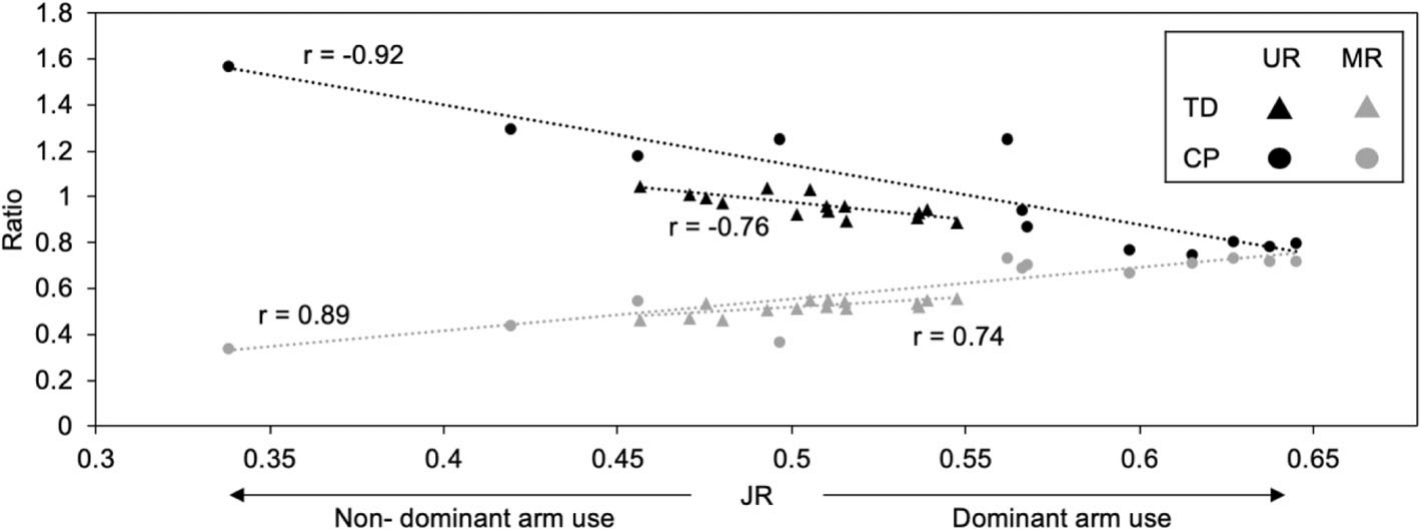
Comparison of JR and activity count-based metrics. Note that as dominant arm use increases, UR decreases, while MR50 increases. Jerk ratio metrics were similar to activity count metrics for both the TD and CP cohorts

The JR was correlated with measures based on activity counts for both TD and CP cohorts (Fig. 3). In the CP cohort, JR correlated with UR and MR with an *r* of −0.92 and 0.89, respectively. In the TD cohort, JR correlated with UR and MR with an *r* of − 0.76 and 0.74. We also evaluated whether similar conclusions could be drawn for ratios calculated from acceleration versus jerk. In contrast to JR, we found that AR was unable to differentiate between the CP and TD cohorts and did not demonstrate changes during therapy (Fig. 1c, f). The AR was also poorly correlated with UR (*r* = 0.52 for CP, *r* = − 0.24 for TD) and MR (*r* = − 0.68 for CP, *r* = 0.44 for TD).

**Fig. 4:**
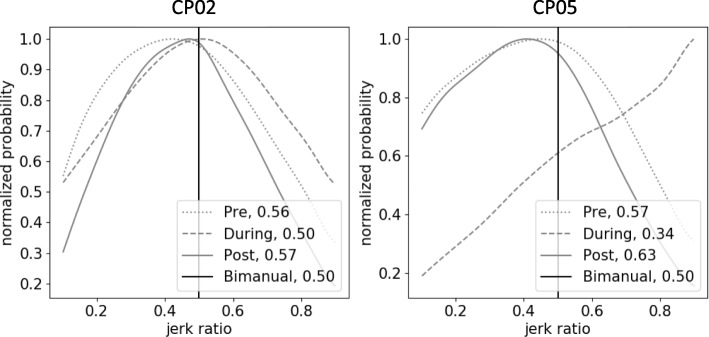
Comparison of JR distributions between two CP participants. Note the difference in shape of distribution during therapy. This distinction might inform clinicians on the participants’ activity level and alignment with therapy goals

While the during-therapy JR distributions all shifted relative to pre- and post-therapy for all five CP participants, the shapes of these distributions showed differences between participants. For example, the during-therapy curve for CP05 increased monotonically while CP02 had a convex distribution during therapy (Fig. 4). The monotonic increase in CP05 suggests that the participant could have been more cognizant about using their non-dominant arm to get the intended practice. Conversely, the convex shape in CP02 indicates that bimanual movement was still present, suggesting that the participant used their restrained arm during therapy.

## Discussion

Calculating JR addresses limitations in contemporary accelerometer metrics by providing a sensitive metric of bimanual arm movement in daily life that avoids the use of activity counts derived from proprietary algorithms. Similar to activity count metrics (UR, MR), we found that the JR differentiated children with CP from TD peers and delineated changes in movement as children with CP participated in CIMT. Furthermore, the consistency of this metric across both collection periods and participants suggest good repeatability. There was also a strong correlation between JR and existing metrics; specifically, UR and MR calculated from the activity counts of one of the most commonly used commercial devices. While one could argue the JR lacks novelty as it resembles existing metrics, its potential lies in the cross-platform comparability as studies using different models of accelerometers could use JR to compare results using a shared, universal unit (*g)*. Furthermore, since the algorithm for this metric is open-source, others can build upon these findings in describing both the quantity and quality of arm motion.

Jerk has become an accepted kinematic metric in evaluating movement of individuals with neurologic disorders since the development of jerk-minimizing models of smoothness in 1985 [20]. Jerk-based metrics have been used with rehabilitation robots to measure smoothness of motion after stroke [28] and to analyze camera-based measures of movement in the home, such as with the Kinect depth camera [29]. While our research explored the potential of jerk to evaluate quantity of movement during daily life and clinical interventions, using jerk measured from wearable sensors to evaluate quality of movement represents an important area for future inquiry. In 2017, Lucena and colleagues derived jerk from a wrist-worn IMU and suggested “jerk asymmetry” and other metrics to understand the correlation between kinematic metrics and existing functional tests. Using principal component analysis to identify the most indicative metric of human motion, they concluded that metrics based on acceleration and jerk contributed to the second principal component and accounted for 31% of variance across nine adult stroke survivors [22]. However, this work relied on an IMU, including both accelerometer and gyroscope, which increases sensor cost and decreases battery life. Our research builds upon this work and demonstrates that accelerometer-derived jerk is repeatable across data collections for TD children and can differentiate movement for a clinical population undergoing occupational therapy for the upper extremity. Our results show that accelerometers alone can produce similar measures to monitor bimanual arm movement in daily life.

Several limitations of the JR need to be considered. In order to capture the daily lives of participants, the collection period in this study was 3-days based upon prior research of individuals with CP [30]. However, due to variations in day-to-day activities, the peak of the JR distribution could vary between data collections. Since we were not focused on the absolute magnitude of JR, but rather the symmetry of the distribution and cumulative probability from JR = 0 to 50, we normalized each distribution by the maximum for comparison between days. Future research should investigate whether jerk magnitude should also be considered in the evaluation of movement intensity. The JR has limited ability in differentiating accelerations caused by rotational or linear movements because it does not use an IMU containing a gyroscope. However, by not focusing on any particular type of motion, this research supports using JR to quantify overall arm movement. While we were able to compare the results to clinical data, only a limited sample size was available for analysis (*n* = 5 in each cohort). The high similarity within the CP and TD cohorts suggests that JR demonstrates unique distributions in these populations that can be used for future comparison. However, future research will be required to investigate the JR distribution of other populations, such as adult stroke survivors or amputees.

To facilitate collaboration among researchers and encourage further development, the algorithm for calculating JR, as well as user-friendly code to produce plots similar to Fig. 1 are provided open-source as Python 3.6 code (Supplementary Material URL: https://steelelab.me.uw.edu/2020/02/jerk-ratio/). With this resource, research groups can use existing or newly created datasets from accelerometers to analyze JR as a complementary metric to existing measures, enabling comparison between research studies or centers that may rely on different sensors and activity count algorithms.

## Conclusion

The JR derived from raw acceleration data captured differences in motion between TD and CP cohorts and across different collection periods before, during, and after therapy. We found that JR correlated with existing activity count metrics including UR and MR that rely on proprietary algorithms. JR was repeatable between data collections for the TD cohort and exhibited high inter-subject similarity within both the TD and CP cohorts. The code for calculating and plotting jerk ratio is open-source and available in the Supplementary Material. By identifying device-independent metrics that can quantify arm movement in daily life, we hope to facilitate collaboration for rehabilitation research using wearable technologies.

## Data Availability

Access to the datasets is restricted to UW researchers and employees of SCH, but algorithms for jerk ratio are open-source. Project name: Jerk Ratio.ipynb Project home page: https://colab.research.google.com/drive/164vnXjduxpuSPt6A0KtO7aISK1Dlwi1I (Shortened one to be provided in proof) Operation system: Platform independent Programming language: Python No license needed.

## Abbreviations

AR: Acceleration ratio
CIMT: Constraint-Induced movement therapy
CP: Cerebral palsy
D: Dominant
IMU: Inertial measurement units
JR: Jerk ratio
MR: Magnitude ratio
ND: Non-dominant
TD: Typically-developing
UR: Use ratio
